# The Role of Metabolites in Cell–Cell Communication: A Review of Databases and Computational Tools

**DOI:** 10.3390/cells15010049

**Published:** 2025-12-26

**Authors:** Qi Song, Zhenchao Liu, Sen Liu

**Affiliations:** 1 Key Laboratory of Fermentation Engineering, Ministry of Education, Cooperative Innovation Center of Industrial Fermentation (Ministry of Education & Hubei Province), Hubei University of Technology, Wuhan 430068, China; 2 Hubei Key Laboratory of Industrial Microbiology, National “111” Center for Cellular Regulation and Molecular Pharmaceutics, Hubei University of Technology, Wuhan 430068, China

**Keywords:** cell–cell communication, metabolite–sensor database, scRNA-seq, polyamine, spatial omics

## Abstract

**Highlights:**

**What are the main findings?**

- The importance of metabolite-mediated cell–cell communication (CCC) has been increasingly recognized recently, yet current metabolite–sensor databases remain inadequate and lack unified data collection standards, which greatly limits the scope of metabolite-mediated CCC that can be analyzed.
- Most current analytical tools follow traditional protein mediated CCC-inference frameworks and therefore struggle to accurately capture the complex interaction mechanisms between metabolites and their sensors. This highlights the need to develop more systematic and accurate inference strategies specifically tailored for metabolite-mediated CCC.

**What are the implications of the main findings?**

- Higher-quality databases and more refined inference frameworks will enable researchers to more accurately understand the communication roles of metabolites in physiological and pathological conditions.
- With the systematic elucidation of metabolite signaling pathways becoming increasingly feasible, the mechanism and key targets underlying specific diseases would become clearer, providing critical insights for drug development and the design of therapeutic strategies.

**Abstract:**

Cell–cell communication (CCC) is essential for multicellular organisms, enabling different cell types to coordinate their activities in both physiological and pathological contexts, such as cell growth, proliferation, tumorigenesis, and immune responses. Metabolites represent an important class of signaling molecules, though their signaling roles were long underappreciated. Growing evidence has highlighted the critical involvement of metabolites in CCC, and the advent of single-cell RNA sequencing (scRNA-seq) has enabled high-resolution exploration of CCC events. This review summarizes existing metabolite–sensor databases and computational tools developed to identify metabolite-mediated CCC using scRNA-seq data. Nonetheless, these databases exhibit considerable variability due to lack of unified collection standards. Most computational tools were adapted from methods used for general CCC inference and often estimate metabolite abundance based on the expression of one or several related genes. Therefore, such approaches are not fully suited to capturing metabolite-mediated CCC due to the complexity of interaction mechanisms between metabolites and their sensors. To address these challenges, improved computational methods and refined databases are needed for the reliable inference of metabolite-mediated CCC. This review discusses the current limitations in database construction and method development, and highlights potential directions for future improvement, including the incorporation of spatial omics and artificial intelligence (AI) approaches. Furthermore, the systematic inference and validation of metabolite-mediated CCC will pave the way for the discovery of novel drugs and therapeutic targets.

## 1. Introduction

Cell–cell communication (CCC) is fundamental to multicellular organisms, orchestrating processes such as metabolic regulation, cell growth, proliferation, tumorigenesis, immune response and other biological processes [1,2,3]. Traditionally, research has focused on protein and peptide ligands—like cytokines and neurotransmitters—that interact with specific receptors to initiate signaling cascades [4,5,6,7,8,9,10]. However, small molecules, particularly metabolites, also serve as crucial signaling entities in CCC [11]. For instance, amino acids like arginine and leucine modulate the mTORC1 pathway by binding to sensors such as CASTOR1 and SAR1A/SAR1B, respectively [12]. Despite their significance, the roles of metabolites in CCC have been underexplored.

The emergence of single-cell RNA sequencing (scRNA-seq), spatial transcriptomics, and other high-throughput technologies has revolutionized our ability to study CCC at unprecedented resolution [13]. These approaches not only enable the systematic identification of ligand–receptor pairs across diverse cell types but also provide new opportunities to infer interactions by integrating transcriptional and spatial information. While protein-mediated CCC has curated ligand–receptor databases and advanced computational tools, such as CellPhoneDB and Cellchat, metabolite-mediated CCC remains underdeveloped, largely due to limited knowledge of metabolite–sensor interactions, where sensors refer to specific receptor proteins, transporters, or intracellular metabolite-binding proteins [14,15]. Moreover, the absence of specialized analytical methods further constrains the systematic investigation of these metabolite-driven signaling networks. However, recent efforts have begun to bridge this gap. For example, MetalinksDB offers extensive catalogs of metabolite–protein interactions, providing a foundation for CCC inference [16]. MEBOCOST utilizes scRNA-seq data to predict metabolite-mediated CCC by integrating knowledge of metabolic enzymes and sensors [17]. Furthermore, frameworks like LIANA+ integrate these databases and methods to incorporate multi-omics and spatial information [18].

Given these advancements, a comprehensive review of current databases and computational tools dedicated to metabolite-mediated CCC is timely and necessary. This review would not only consolidate existing knowledge but also identify prevailing challenges and guide future research directions, ultimately deepening our understanding of metabolite-driven intercellular communication in both physiological and pathological contexts.

## 2. Metabolite-Mediated Cell Communication

### 2.1. Signaling Metabolites: Concept and Definition

Metabolites have traditionally been regarded mainly as substrates or products to fulfill cellular demands. Yet, their roles as signaling molecules have also long been appreciated. Classical signaling systems include hormones and neurotransmitters, among which metabolite-derived or metabolite-like messengers—such as dopamine and GABA—play indispensable roles [19,20]. In addition, primary metabolites within the fundamental metabolic pathway can themselves function as signaling cues by activating or inhibiting specific enzymes to maintain homoeostasis. An example is provided by ADP and ATP: fluctuations in their intracellular levels regulate the activity of phosphofructokinase-1 (PFK1), thereby maintaining energy homeostasis [21,22]. Baker et al. attempted to propose criteria that distinguish signaling metabolites from hormones [11]. A useful perspective is that, under physiological or pathological conditions, a signaling metabolite should maintain a measurable concentration in the extracellular space, while the abundance of its precursor fluctuates within a defined range. Moreover, the metabolite itself should have clearly established intracellular metabolic roles and physiological functions.

### 2.2. Physiological and Pathological Roles of Signaling Metabolites

A major role of classical metabolic signals is to maintain metabolic homeostasis via conveying information about nutrient availability, energetic status, and environmental conditions. Through this signaling capacity, physiological balance is maintained via coordinated downstream transcriptional, translational, and other multi-layered regulatory mechanisms. Glycolysis and gluconeogenesis provide a well-established example of this principle, as these pathways are subject to complex, multi-level regulation to ensure metabolic stability. Cellular energy status, reflected by ATP, ADP, and AMP levels, is sensed by key enzymes such as PFK1 to fine-tune metabolic flux [21,22]. In addition, these metabolic processes are tightly integrated with hormonal signaling; for instance, insulin increases intracellular fructose-2,6-bisphosphate (F2,6BP) levels by regulating PFK2 activity, and F2,6BP in turn potently activates PFK1 [23]. In contrast, key enzymes involved in fatty acid catabolism are inhibited by fatty acyl–CoAs (FA-CoAs), while insulin signaling suppresses fatty acid catabolism by inhibiting carnitine palmitoyl-transferase 1 (CPT1), even when fatty acyl–CoAs are present at relatively high abundance [24].

On the other hand, in pathological contexts, dysregulation of metabolic signaling often represents a key driving force in disease initiation and progression. For instance, amino acid signaling—particularly involving leucine, arginine, and glutamine—plays a critical role in regulating mTORC1 activity. mTORC1 serves as a central signaling hub that integrates nutrient availability to control cell growth and protein synthesis, and it is frequently hyperactivated in cancer [25]. Arginine binding to CASTOR1 and leucine binding to SESTRIN2/1 lead to the release of GATOR2 and subsequent activation of the mTORC1 signaling pathway [26,27,28]. Lactate is another metabolite that has attracted increasing attention in recent years in various diseases including cancer [29]. For example, regulatory T cells can uptake lactate within the tumor microenvironment, where lactate signaling through MOESIN enhances TGF-β signaling, reinforcing an immunosuppressive phenotype in tumors [30]. Similarly, phosphoenolpyruvate (PEP) has also been identified as a critical signaling molecule in T cells; reduced PEP levels impair T cell effector function by diminishing cytoplasmic Ca^2+^ [31]. Collectively, these findings highlight the critical regulatory roles of signaling metabolites under both physiological and pathological conditions.

### 2.3. Signaling Metabolites Across Biological Systems

Beyond the roles in signal transmission within individual organisms, metabolites have long been recognized as mediators of communication across distinct biological entities. A prominent and extensively studied example is the crosstalk between the gut microbiota and the host [32,33]. The gut provides a nutrient- and energy-rich environment for microorganisms, while microbial-derived metabolites, in turn, act as key signaling molecules that shape host homeostasis. For example, accumulating evidence demonstrates that short-chain fatty acids (SCFAs) modulate immune cell function and exert anti-inflammatory effects, thereby playing an essential role in maintaining intestinal homeostasis [34,35,36]. In parallel, host-derived primary bile acids are modified by gut microbiota to generate secondary bile acids, which engage host receptors such as FXR and TGR5 to regulate energy metabolism and immune function [37,38]. Moreover, microbial-derived indole signals through the aryl hydrocarbon receptor (AhR), contributing to the maintenance of mucosal immunity and intestinal barrier integrity [39,40].

In addition to microbiota–host interactions, signaling metabolites also play central roles in coordinating development and environmental adaptation microbe–plant system. Root exudates provide the foundation for plant–microbe symbiosis and actively shape the rhizosphere by recruiting beneficial microorganisms. In turn, many microbial-derived metabolites function as critical signaling cues that regulate plant growth and stress responses, including hormones such as indole-3-acetic acid (IAA) and gibberellins (GA) [41,42,43]. In addition, although the underlying mechanisms are not yet fully elucidated, a large number of evidence have indicated that several microbial metabolites exert pronounced effects on plant disease resistance and stress tolerance, exemplified by lipopeptides such as iturin, surfactin, and fengycin [44,45]. Collectively, these observations underscore the important roles of metabolites as information carriers mediating intercellular communication across diverse biological systems.

### 2.4. Mechanistic Basis of Metabolite-Mediated CCC

**Figure 1:**
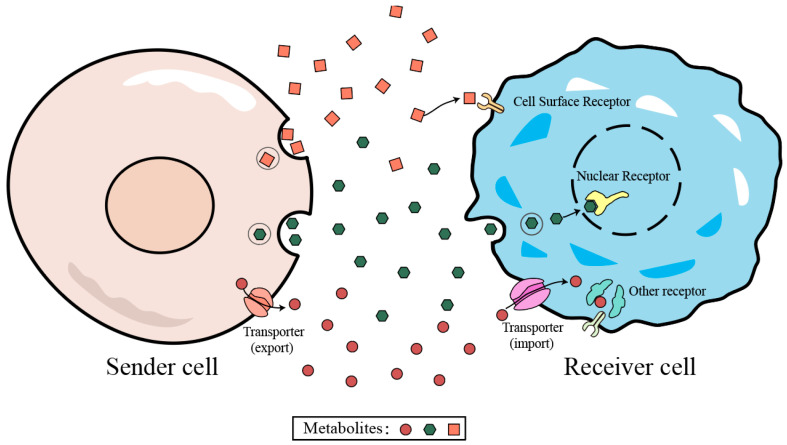
Models of metabolites-mediated CCC via metabolite–sensor interactions.

In metabolite-mediated CCC, the release of metabolites from one cell and their subsequent uptake or sensing by another cell constitutes a fundamental mechanism [11,17,46]. Accordingly, transport represents a critical determinant of metabolite-mediated signaling. Unlike many protein ligands, which typically rely on well-defined secretion and trafficking pathways, the mechanisms governing metabolite transport remain less characterized. Metabolites may be released through exocytosis or via membrane transporters, although these processes have broad substrate specificity and can mediate the export of various metabolites. However, metabolites often have different chemical modifications, increasing the complexity of the export process. Polyamines provide a representative example [47,48]. Their release from the intracellular space into the extracellular milieu occurs mainly through two mechanisms (Figure 1). One route entails plasma membrane export mediated mainly by solute carrier (SLC) transporters. For instance, SLC3A2 is implicated in forming a complex with spermidine/spermine N1-acetyltransferase 1 (SAT1), thereby facilitating the export of acetylated polyamines [49,50]. Alternatively, polyamine release could occur via vesicle-mediated exocytosis, in which polyamines are packaged into intracellular vesicles and secreted into the extracellular space [51].

The sensing of metabolic signals can likewise occur through multiple mechanisms, among which transport remains a central component (Figure 1). Although most currently identified metabolite receptors are cell surface proteins, metabolite recognition or action may also occur on the cytosolic side of the cell. Polyamines again provide an illustrative example. Extracellular spermine (SPM) has been shown to interact with the calcium-sensing receptor (CaSR) and modulate its activity [52]. In parallel, polyamines can also enter cells through transporter-mediated uptake, with several solute carrier proteins, including SLC22A1 and SLC22A4, having been reported to participate in this process, or via endocytic pathways facilitated by molecules such as glypican-1 (GPC1) and caveolin-1 (CAV1) [53,54,55,56]. Once inside the cell, polyamines exert diverse regulatory effects, including inhibition of ion channels such as KCNJ4 and TRPM4 [57,58]. In addition, intracellular spermidine can directly bind to JAK1, regulating immune-related functions by restricting the binding of receptor proteins to cytokines [59]. This illustrates the tight functional coupling between metabolite sensing and other receptor-dependent signaling pathways [11].

## 3. Experimental and Integrative Approaches for Studying Metabolite-Mediated Cell–Cell Communication

To systematically dissect the complex network of metabolite-mediated CCC, researchers have progressively developed and integrated a broad array of experimental and computational approaches [1]. Among experimental strategies, high-resolution mass spectrometry–based metabolomics and proteomics provide a foundational platform for the qualitative and quantitative profiling of metabolites and proteins, enabling the characterization of cell type–specific metabolic signatures, receptor expression, and their spatial distributions within tissues [60,61,62]. The application of stable isotope labeling further allows the tracing of metabolic fluxes and reveals the dynamic transport of metabolites between interacting cell populations [63]. In addition, in vitro co-culture systems and microfluidic platforms offer versatile and well-controlled experimental frameworks for validating the functional roles of metabolites in CCC under defined conditions [64,65,66]. In recent years, emerging single-cell technologies, such as scRNA-seq and spatial transcriptomics, have laid the foundation for systematically characterizing metabolite-mediated CCC at single-cell resolution [67,68]. Consequently, integrative multi-omics strategies have become central to elucidating the underlying mechanisms of metabolite-mediated CCC. However, the accuracy and throughput of metabolite and protein measurements at the single-cell scale remain limited. As a result, inferring metabolite abundance from gene expression profiles currently represents a pragmatic compromise.

## 4. Databases and Tools of Metabolite-Mediated CCC Inference

Based on scRNA-seq and spatial transcriptomic data, several computational methods and software tools have been developed to systematically analyze metabolite-mediated CCC [69]. These approaches typically rely on prior knowledge of metabolite–sensor pairs, and the availability of well-curated metabolite–sensor databases is therefore critical for the reliable inference of CCC events from scRNA-seq data. Here, we collected and organized metabolite–sensor interactions from six recent databases, most of which were embedded in their corresponding computational tools (Table 1) [14,16,17,70,71,72]. To ensure data consistency across databases, each metabolite were annotated with HMDB ID, and each sensor were labeled using HGNC symbol [73,74]. Records from the databases with HMDB ID or gene symbol missing were excluded. In addition, if some databases contain interactions from several species, only those of human were considered.

### 4.1. CellPhoneDB

CellPhoneDB is a classic database and tool designed to infer CCC from scRNA-seq data, which is widely used since its first release in 2018 [14]. In the previous version, CellPhoneDB mainly focuses on protein ligand–receptor analysis, only a few metabolite–sensor partners were included. Compared to previous versions, CellPhoneDBv5 further enhances downstream inference by considering the role of metabolite transporters and collecting more sensors from specific sources. In addition, it accounts for the subunit architecture of ligands or receptors, and hence one metabolite–sensor pair could have several cofactors. In our statistics, these records were combined into ones. CellPhoneDBv5 obtained and organized metabolite–sensor data from Reactome, HMRbase and GPCRdb and Rhea, containing 217 metabolite–sensor interactions of 47 metabolites and 155 sensors (Table 1) [75,76,77,78]. In the algorithm of inferring CCC, estimation of metabolite level is a key issue. Compared to protein ligand, the levels of metabolites are regulated by a broader range of factors and exhibit a more complex relationship with related gene expressions. CellPhoneDB estimates metabolite concentrations by using the expression level of the last enzyme in its biosynthesis and known transporter.

### 4.2. NeuronChatDB

NeuronChat is a specialized tool designed for studying neural communication, and non-peptide ligands such as metabolites are a vital components, accounting for more than a half ligands of its neural-specific database—NeuronChatDB [72]. NeuronChatDB is manually well curated from KEGG, IUPHAR/BPS Guide to PHARMACOLOGY(GtoPdb), and relevant literature, including 126 interactions of 10 metabolites and 105 sensors (Table 1) [79,80]. On the inference algorithm side, NeuronChat also takes the enzymes in synthesis and vesicular transporters into consideration. But it grouped the genes with functions, using the arithmetic mean to summarize genes in the same group since the genes are redundant for the same function, and the geometric mean to combine across different groups.

### 4.3. scConnect

scConnect obtained and organized data from GtoPdb, including ligand–receptor and metabolite–sensor pairs [70,79]. Following the method of Zeisel et al. (2018), metabolites dependent on several sets of genes were manually collected and organized [81]. In addition, scConnect extends the data by incorporating orthologous genes, under the assumption that ortholog proteins also interact with their ligands. Finally, there are 572 metabolite–sensor interactions of 230 metabolites and 283 sensors in the database of scConnect (Table 1). In inferring CCC, scConnect considers not only one or more biosynthetic enzymes but also incorporates both vesicular and reuptake transporters as essential components. Furthermore, it explicitly excludes enzymes responsible for converting a given ligand into another metabolite, ensuring specificity in the predicted signaling interactions.

### 4.4. MEBOCOST

MEBOCOST concentrates on resolving metabolite-mediated CCC inference and collected data from specific databases, including Recon2, GPCRdb and some other well-known databases [17,73,77,82,83,84]. In addition, MEBOCOST classifies sensors into cell surface receptors, nuclear receptors and transporters, and collected data from specific databases, including NicheNet, UniProt, Transporter Classification Database (TCDB), Nuclear Receptor Signaling Atlas (NURSA) [85,86,87]. With lists of extracellular metabolites and sensors, MEBOCOST creates a text-mining method based on PubMed abstracts, obtaining curated metabolite–sensor partners. In total, it contains 432 metabolite–sensor interactions of 157 metabolites and 248 sensors (Table 1). To estimate the presence of metabolite, MEBOCOST considers genes related to both its accumulation and consumption. In addition, MEBOCOST attempted to improve metabolite level estimation by using flux balance analysis (FBA) tools, such as scFEA and COMPASS, but it did not yield superior performance compared to simpler methods, which were adopted as the primary method finally [88,89,90,91]. Hence, a simplified formula was adopted to reduce computation time without losing accuracy. On the other hand, the diversity of metabolite sensors—encompassing receptors and transporters across various cellular locations—increases the complexity of CCC inference. In the future, new inference algorithms could improve performance by modifying FBA method and concerning the difference of interaction among sensor types.

### 4.5. MRCLinkDB

Before developing MRCLinkDB, the team created Cellinker, a widely known ligand–receptor database, already including hundreds of non-peptide ligands [92]. Build on this foundation, MRCLinkDB continually compiled data from literature and established databases, including GtoPdb, KEGG and MEBOCOST [17,79,80]. Moreover, each recorded metabolite–sensor interaction is backed by experimental evidence, ensuring its reliability. It contains 680 metabolite–sensor interactions, encompassing 186 metabolites and 388 sensors (Table 1). In addition, MRCLinkDB built a webserver to store these collected data and recent published tools and offer service of metabolite-mediated CCC inference online from scRNA-seq data. It estimates metabolite concentration by combining the expression levels of synthesis and degradation enzymes and incorporating the presence of specific transport proteins.

### 4.6. MetalinksDB and LIANA+

MetalinksDB integrated several prior databases, such as CellphoneDB, NeuronChat, Cellinker, scConnect, STITCH and some other resources [14,70,72,76,92,93]. Additionally, it annotates the mode of action for each interaction based on STITCH. MetalinksDB supports flexible data retrieval through a web interface, allowing users to filter by confidence level, tissue location and other criteria. For instance, confidence levels are assigned to interactions depending on the source database, experiment evidence and other factors. The database also provides code scripts for data updating. In total, MetalinksDB contains a relatively large number of metabolite–sensor interactions, reaching 10,242 interactions of 497 metabolites and 769 sensors (Table 1). To ensure the reliability of inference, it is necessary to apply cautious threshold filtering before use in practice. To infer CCC events, the group launched LIANA+, a comprehensive framework containing multiple existing inference algorithms [18]. Customed MetalinksDB served as its built-in database for metabolite-mediated CCC inference [16]. In addition, a significant feature of LIANA+ is it includes multi-omics data, particularly spatial omics, in CCC inference. Since CCC occurs within defined microenvironments, incorporating spatial co-localization data can remarkably reduce the false positives.

### 4.7. Comparative Analysis of Existing Databases

Among the six databases and tools mentioned above, NeuronChat contains only a limited number of neuron-specific metabolites and sensors, most of which are already included in other resources. Therefore, our subsequent analyses focused on the remaining five databases. As CellPhoneDB and scConnect are comprehensive CCC analysis tools, metabolite–sensor information constitutes only a minor portion of their interaction datasets. Hence, the number of metabolite-related entries in these tools is limited and the mixed storage of different data types makes metabolite-specific information less accessible. In contrast, MEBOCOST, MRCLinkDB and MetalinksDB specifically target metabolite-mediated CCC. Both MRCLinkDB and MetalinksDB offer web servers that facilitate convenient data access for users.

**Figure 2:**
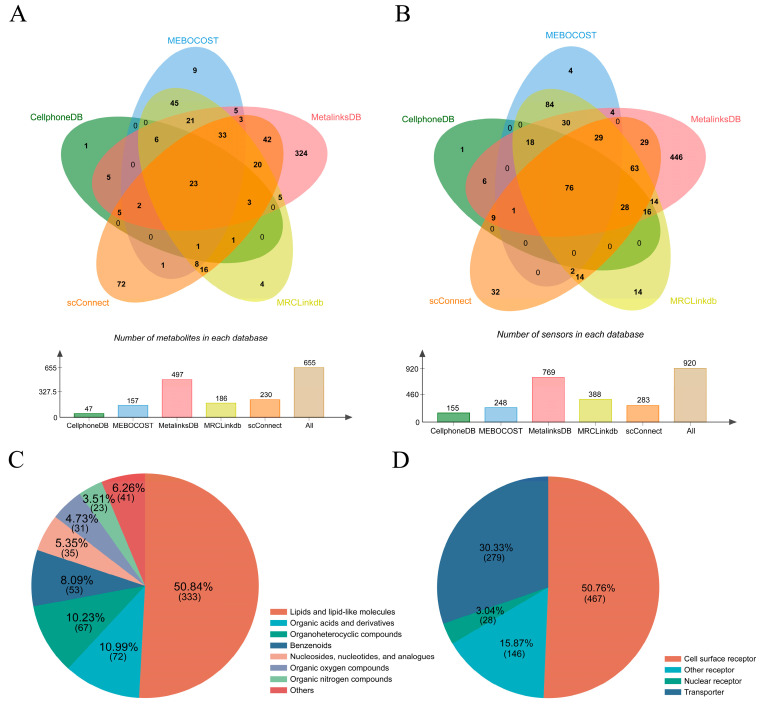
Classification of metabolites and sensors in the databases. (**A**): Comparison of metabolites in five databases; (**B**): Comparison of sensors in five databases; (**C**): Classifications of metabolites collected from five databases; (**D**): Classifications of sensors collected from five databases.

Although these databases have similar data sources, our statistical comparison revealed that each database contains its own distinct set of metabolites and sensors (Figure 2A,B). MetalinksDB employs specific strategies to curate a substantially larger set of metabolite and sensors than the other databases, interactions involving certain metabolites, such as polyamines, are still underrepresented. This limitation is partly attributable to the exclusion of metabolites that are not annotated as “extracellular” in HMDB, a criterion that may not reliably reflect whether a metabolite is secreted [16]. The lack of a unified and credible data curation standard is another limit, as a considerable amount of relevant information remains dispersed across the literature and has not yet been incorporated into existing databases. Combined five databases, lipids and lipid-like molecules constitute the predominant class of signaling metabolites, accounting for 50.84% of the 655 metabolites cataloged. Other significant categories include organic acids and derivatives, organoheterocyclic compounds, benzenoids, organic oxygen compounds, and organic nitrogen compounds (Figure 2C and Table S1). Regarding sensors, a total of 920 proteins were compiled from these databases. Among them, 50.76% are cell surface receptors, 3.04% are nuclear receptors, and 15.87% are receptors located in other subcellular compartments. Transporters represent another crucial category, comprising 30.33% of the total sensors (Figure 2D, Table S2).

**Figure 3:**
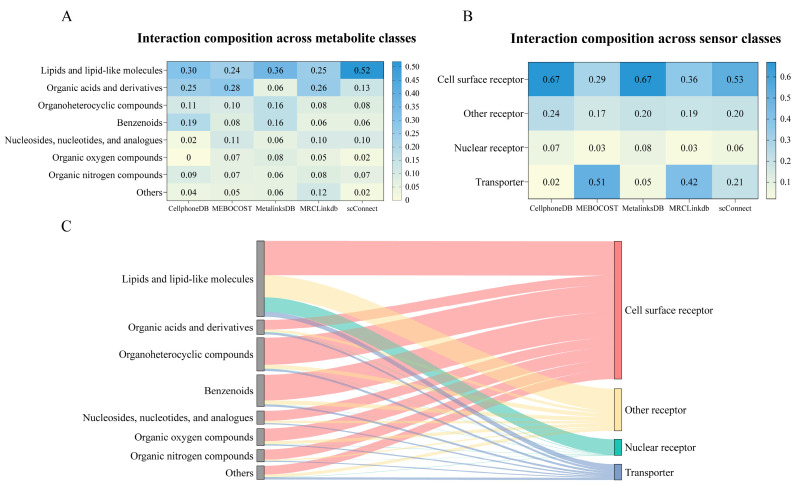
Composition of metabolite–sensor interactions across the five databases. (**A**): Composition of interactions by metabolite classes across the five databases; (**B**): Composition of interactions by sensor classes across the five databases; (**C**): Metabolite–sensor interactions across metabolite and sensor classes in combined data.

With respect to interactions, the more comprehensive curation strategy adopted by MetalinksDB results in a substantially larger number of interactions. As interactions categorized by metabolite class, the overall distribution is consistent across the databases, with lipid-mediated CCC representing the largest proportion (Figure 3A). In contrast, classification by sensor type reveals notable differences: MEBOCOST and MRClinkDB exhibit a higher proportion of transporter-associated interactions, which likely reflect differences in data collection strategies and curation preferences (Figure 3B). Notably, although transporter-related interactions account for a relatively smaller proportion in MetalinksDB, its large dataset size means that the absolute number of transporter-related interactions is still among the highest across databases. Finally, analysis of the combined dataset illustrates the current landscape of metabolite–sensor interactions across different metabolite and sensor classes, highlighting cell-surface receptors as the predominant category (Figure 3C and Table S3). As interaction data curation strategies continue to improve and datasets expand, biases introduced by data collection preferences are expected to be progressively mitigated.

## 5. Conclusions and Discussions

Metabolites play pivotal roles in both physiological and pathological conditions by serving not only as energy sources but also as signaling molecules and modulators of diverse biological processes. Metabolite-mediated CCC constitutes a critical regulatory layer that shapes cellular behavior across tissues. With the advent of scRNA-seq and the emergence of metabolite–sensor databases, the systematic identification of metabolite-mediated CCC becomes important and feasible. Multiple scRNA-seq based CCC inference tools have been developed, substantially advancing our understanding of cellular heterogeneity and intercellular communication. However, a bottleneck limiting the broader application of current approaches is the lack of high-quality, standardized databases.

In this context, MetalinksDB provides substantial support by offering extensively curated collections of both enzyme–transporter–metabolite and metabolite–sensor datasets [16]. Moreover, it introduces a strategy for constructing tissue- and disease-specific metabolite–sensor datasets and assigning credibility levels to curated interactions, with the aim of improving inference reliability. Nevertheless, several aspects of this framework need further consideration. Firstly, MetalinksDB excludes metabolites that are not annotated as “extracellular” in HMDB, which may result in the unintended omission of biologically important metabolites. Secondly, the construction of sample-specific metabolite and sensor databases might be too stringent for the discovery of novel interactions. A robust strategy would be to establish a comprehensive reference database and subsequently filter implausible interactions based on sequencing or experimental data, for example, by excluding interactions involving metabolites or receptors with insufficient abundance in extracellular space. In summary, as databases continue to improve, benchmarking across tools is expected to become more objective, thereby facilitating the development of more efficient and accurate analytical methods.

## 6. Future Perspectives

The rapid advancement of single-cell multi-omics has enabled high-resolution inference of metabolite-mediated CCC [68]. Although transcriptomic data and FBA-based approaches could provide indirect estimates of metabolite abundance, MS-based measurements remain essential in driving the development of highly sensitive and spatially resolved metabolomics technologies, particularly at single-cell and spatially resolved scales. In parallel, the integration of AI-driven inference methods is expected to further enhance the predictive power and scalability of CCC analyses. Moreover, the remarkable diversity of metabolite-mediated CCC mechanisms across biological systems highlights the need for context-specific analytical frameworks, encompassing interactions such as host–microbe and microbe–plant crosstalk. Continued advances in both experimental and computational methodologies will deepen our understanding of metabolite-mediated CCC and facilitate the development of novel diagnostic and therapeutic strategies.

## Figures and Tables

**Table 1:** Databases containing metabolite–sensor interactions.

Tool and Database	Release Year (Version)	Platforms/Programing Language	Input	Description	Repository	Metabolite–Sensor Interaction	Metabolites	Sensors	Data Sources	Country
CellPhoneDB	2023 (v5.0.0)	Python	scRNA-seq	Using the last representative enzyme as a proxy of non-peptidic ligand abundance	https://github.com/ventolab/CellphoneDB (accessed on 9 April 2025)	217	47	155	Reactome, HMRbase, GPCRdb and Rhea	UK
NeuronChat (NeuronChatDB)	2023	R	scRNA-seq; Spatial data	Neural-specific CCC inference	https://github.com/Wei-BioMath/NeuronChat (accessed on 3 May 2025)	126	10	105	KEGG, GtoPdb and studies	USA
scConnect	2021 (v1.0.4)	Python	scRNA-seq	Taking enzymes of synthesis and transporters into metabolite-mediated CCC inferences	https://github.com/JonETJakobsson/scConnect (accessed on 1 May 2025)	572	230	283	GtoPdb and studies	Sweden
MEBOCOST	2024 (v1.0.4)	Python	scRNA-seq	Specific to infer metabolite-mediated CCC events	https://github.com/zhengrongbin/MEBOCOST (accessed on 18 April 2025)	432	157	248	HMDB, Recon2, GPCRdb, Wikipedia, GeneCards and studies	USA
MRClinkDB	2024	Web	scRNA-seq	User-friendly Web interface	https://www.cellknowledge.com.cn/mrclinkdb (accessed on 6 May 2025)	680	186	388	GtoPdb, MEBOCOST, KEGG and studies	China
Liana+ (MetalinksDB)	2025 (v1.5.1)	Python	scRNA-seq; Spatial data	Framework including existing methods and databases with multi-omics	https://github.com/saezlab/liana-py; https://metalinks.omnipathdb.org/ (accessed on 23 April 2025)	10,242	497	769	CellphoneDB, NeuronChat, Cellinker, scConnect, STITCH, Rhea and studies	Germany

## Data Availability

The original contributions presented in this study are included in the article. Further inquiries can be directed to the corresponding authors.

## Supplementary Materials

The following supporting information can be downloaded at: https://www.mdpi.com/article/10.3390/cells15010049/s1, Table S1: Metabolites collected from the five databases and their classification in HMDB; Table S2: Sensors collected from the five databases and their classification; Table S3: Interactions collected from the five databases and their classification.

## Abbreviations

CCC	Cell–cell communication
scRNA-seq	Single-cell RNA sequencing
AI	Artificial intelligence
PFK1	Phosphofructokinase-1
F2,6BP	Fructose-2,6-bisphosphate
FA-CoA	Fatty acyl–CoA
CPT1	Carnitine palmitoyl-transferase 1
PEP	Phosphoenolpyruvate
SCFA	Short-chain fatty acid
AhR	aryl hydrocarbon receptor
IAA	indole-3-acetic acid
GA	Gibberellins
SLC	Solute carrier
SAT1	Spermidine/spermine N1-acetyltransferase 1
SPM	Spermine
CaSR	Calcium-sensing receptor
GPC1	Glypican-1
CAV1	Caveolin-1
GtoPdb	IUPHAR/BPS Guide to PHARMACOLOGY
TCDB	Transporter Classification Database
NURSA	Nuclear Receptor Signaling Atlas
FBA	Flux balance analysis
